# Effects of dietary supplementation with 3-nitrooxypropanol on enteric methane production, rumen fermentation, and performance in young growing beef cattle offered a 50:50 forage:concentrate diet

**DOI:** 10.1093/jas/skad399

**Published:** 2023-12-01

**Authors:** Stuart F Kirwan, Luis F M Tamassia, Nicola D Walker, Alexios Karagiannis, Maik Kindermann, Sinéad M Waters

**Affiliations:** Animal Bioscience Research Department, Teagasc Grange, Dunsany, County Meath, Ireland C15 PW93; DSM Nutritional Products, Animal Nutrition and Health, Wurmisweg 576, 4303 Kaiseraugst, Switzerland; DSM Nutritional Products, Animal Nutrition and Health, Wurmisweg 576, 4303 Kaiseraugst, Switzerland; DSM Nutritional Products, Animal Nutrition and Health, Wurmisweg 576, 4303 Kaiseraugst, Switzerland; DSM Nutritional Products, Animal Nutrition and Health, Wurmisweg 576, 4303 Kaiseraugst, Switzerland; Animal Bioscience Research Department, Teagasc Grange, Dunsany, County Meath, Ireland C15 PW93

**Keywords:** beef cattle, enteric methane, greenhouse emissions, methane inhibitor, 3-nitrooxypropanol

## Abstract

There is an urgent requirement internationally to reduce enteric methane (CH_4_) emissions from ruminants to meet greenhouse gas emissions reduction targets. Dietary supplementation with feed additives is one possible strategy under investigation as an effective solution. The effects of the CH_4_ inhibitor 3-nitrooxypropanol (3-NOP) at reducing CH_4_ emissions in beef have been shown mainly in adult cattle consuming backgrounding and high-energy finishing diets. In this study, the effects of dietary supplementation of young growing (≤6 mo) beef cattle with 3-NOP were examined in a 50:50 forage:concentrate diet. A total of 68 Dairy × Beef (Aberdeen Angus and Hereford dairy cross) male calves (≤6 mo of age at the start of experiment, body weight: 147 ± 38 kg) underwent a 3-wk acclimatization period and were then assigned to one of two treatments in a completely randomized block design. Dietary treatments were (1) control, placebo (no 3-NOP), and (2) 3-NOP applied at 150 mg kg^−1^ DM. Calves were fed a partial mixed ration for 12 wk. Body weight was recorded weekly and feed intake daily using the Calan Broadbent feeding system. Methane and hydrogen emissions were measured using the GreenFeed system. Total weight gained, dry matter intake (DMI), and average daily gain were not affected by 3-NOP (*P* > 0.05) supplementation. On average, the inclusion of 3-NOP decreased (*P* < 0.001) CH_4_ emissions: g d^−1^; g kg^−1^ DMI; by 30.6% and 27.2%, respectively, during the study with a greater reduction occurring over time. Incorporating 3-NOP into beef cattle diets is an efficient solution to decrease CH_4_ emissions during indoor feeding and when offered 50:50 forage:concentrate diet.

## Teaser

This research highlights the efficacy of the methane (CH_4_) inhibitor, 3-nitrooxypropanol, in reducing CH_4_ emissions in young growing beef cattle without affecting dry matter intake and animal performance when consuming a 50:50 forage:concentrate diet.

## Introduction

Methane (CH_4_) is a major greenhouse gas (GHG) with a global warming potential 28 times that of carbon dioxide (CO_2_) (Pörtner et al., 2022). Globally, enteric CH_4_ accounts for 4% to 6% of anthropogenic GHG emissions (Gerber et al., 2013). The Paris Agreement is a legally binding international treaty, which aims to limit global warming potential to well below 2 °C and preferably 1.5 °C. The European Green Deal has set out to reduce GHG emissions by 55% (compared to 1,990 levels) by 2,030 and achieve climate neutrality by 2,050. In Ireland, agriculture accounts for more than 37% of Irish GHG emissions (DECC, 2021) of which ~60% comprises of CH_4_ emissions (EPA, 2021).

Enteric CH_4_ production results from the anaerobic fermentation of organic matter in the rumen by bacteria, protozoa, and fungi, which produces hydrogen (H_2_) and CO_2_. The formation of CH_4_ by methanogenic archaea from CO_2_ and H_2_ is a major pathway for the removal of H_2_ from the system (Haque, 2018). In addition to the negative impact on the environment, the formation of enteric CH_4_ represents 2% to 12% loss of gross energy of total feed intake (Johnson and Johnson, 1995).

A comprehensive range of solutions have been identified as possible mitigation strategies to reduce enteric CH_4_ emissions from ruminants (Hristov et al., 2013; Beauchemin et al., 2020). The CH_4_ inhibitor 3-nitrooxypropanol (3-NOP; DSM Nutritional Products Ltd., Kaiseraugst, Switzerland) has exhibited considerable potential in reducing enteric CH_4_ production in the range of 20% to 80% from ruminants. The compound targets the nickel enzyme methyl-coenzyme M reductase in the final step in the formation of CH_4_ by rumen archaea (Duin et al., 2016). Previous studies have indicated that 3-NOP is effective in reducing enteric CH_4_ emissions in dairy cattle (Van Wesemael et al., 2019; Melgar et al., 2020a, 2020b, 2020c; Schilde et al., 2021), beef cattle (Romero-Perez et al., 2015; Vyas et al., 2016, 2018; Kim et al., 2019; McGinn et al., 2019), and sheep (Martínez-Fernández et al., 2014). However, the level of reductions in CH_4_ attained when supplementing with 3-NOP depends on a number of factors: type of animal, diet, and method of supplementation (Dijkstra et al., 2018). Conversely, reductions in feed intake were observed in a number of studies when 3-NOP was offered at 200 mg kg^−1^ dry matter intake (DMI) in backgrounding beef cattle diets without any negative effects on performance (Romero-Perez et al., 2014; Vyas et al., 2016), while having no effect on intake when offered in finishing diets (Vyas et al., 2018). Equally, when offered at 330 mg kg^−1^ DMI to cattle consuming an ad libitum forage diet (Martinez-Fernandez et al., 2018), there was no difference in DMI during the dietary adaptation phase, however, when the animals moved into the open-circuit respiration chambers DMI increased while reducing CH_4_ emissions. Furthermore, the effectiveness of 3-NOP in reducing CH_4_ emissions from beef cattle has mainly focused on cattle-fed backgrounding diets with approximate body weights (BW) of 300 kg (Alemu et al., 2021) and finishing diets of approximately 600 kg BW (Vyas et al., 2016).

Meale et al. (2021) investigated the effects of early life intervention with 3-NOP via an oral gavage ~2 h post feeding, in dairy calves from birth until 3 wk postweaning. While treated calves exhibiting a persistent reduction in CH_4_ emissions postweaning until 12-mo age, there is little information available on the efficacy of dietary supplementation of solid feed with 3-NOP in young growing beef cattle (<6 mo, <150 kg BW). It is hypothesized that the inclusion of 3-NOP will inhibit methanogenesis without affecting DMI and has no adverse effect on animal performance in young growing cattle. Therefore, the objective of this study was to evaluate the effects of 3-NOP inclusion on enteric CH_4_ production, rumen fermentation, and performance in young growing beef cattle offered a 50:50 forage–concentrate diet.

## Materials and Methods

All animal procedures used in this study were approved by the Teagasc Animal Ethics Committee and conducted using procedures consistent with the experimental license (AE19132/P136) issued by the Irish Health Products Regulatory Authority in accordance with European Union legislation (Directive 2010/63/EU), for the protection of animals used for scientific purposes.

### Animals and experimental design

A total of 68 Dairy × Beef male calves (Aberdeen Angus and Hereford dairy cross) (147 ± 38 kg) were blocked on BW at the beginning of the experiment and assigned to one of two treatments (*n* = 34). For the duration of the study, animals were accommodated indoors in one of four slatted floor pens, with 17 animals per pen (3.72 m^2^ animal^−1^); pens were balanced for BW.

The experimental design was a randomized complete block design with a 3-wk covariate period at the start of the study, followed by a 12-wk experimental period. Following the covariate period, the animals were blocked in 34 blocks of two, based on their BW, breed, and enteric CH_4_ emissions. Bulls within the block were randomly assigned to one of two treatments: (1) control, placebo (no 3-NOP) and (2) 3-NOP applied at 150 mg kg^−1^ on a dry matter (DM) basis.

Dietary treatments were offered as a partial mixed ration (PMR) once daily at 0900 hours, and all animals had free access to clean drinking water. For the duration of the experiment, dietary treatments were offered ad libitum (110% of previous day’s intake) which consisted of grass silage (GS), barley straw, and concentrate (Table 1). Animals received their respective diets using a Calan Broadbent controlled feeding system (American Calan, Northwood, NH, USA) to facilitate the recording of individual DMI. The composition of the PMR was the same during both covariate and experimental periods. The basal PMR was prepared daily using a mobile farm mixer (Alltech Keenan: Keenan MechFiber320; MF32M128). To avoid any cross contamination, separate feed mixers were used to mix the control and 3-NOP PMR. The placebo and 3-NOP supplement were incorporated into the PMR (Table 1), through a premix containing (%, fresh weight basis): 5% soyabean oil, 65% ground maize meal, and 30% of 3-NOP or placebo supplement (DSM Nutritional Products, Basel, Switzerland), prepared 2 d prior to feeding, kept at 4 °C in sealed containers, and mixed daily with the PMR before feeding. The active supplement contained 10.9% 3-NOP on a carrier of silicone dioxide (SiO_2_) and propylene glycol; the placebo supplement contained SiO_2_ and propylene glycol only.

**Table 1.** Ingredient inclusion rate and composition (mean ± SD) of basal PMR diet

Item	
Ingredient, % DM	
Grass silage^1^	45.0
Concentrate^2^^,^^3^	50.0
Barley straw	5.0
Chemical composition, % DM	
Dry matter, % as fed	40.89 ± 3.54
Crude protein	17.71 ± 1.54
Starch	18.88 ± 3.04
Neutral detergent fiber	36.21 ± 5.29
Acid detergent fiber	28.81 ± 4.30
Ash	8.40 ± 0.49
Ether extract	2.99 ± 0.04
Gross energy (MJ/kg DM)	17.63 ± 0.51

^1^Grass silage analysis on a DM basis: crude protein 11.9%, neutral detergent fiber 45.3%, acid detergent fiber 26.9%, ash 8.8%, and ether extract 3.4%.

^2^Composition of concentrate on a DM basis: 30% rolled barley, 20% maize meal, 21.59% soyabean meal, 10.59% soy hulls, 10% distillers grains, 5% molasses, 1.64% calcium carbonate, 0.67% Salt, 0.5% mineral and vitamin premix.

^3^Concentrate analysis: crude protein 25.2%, starch 46.0%, neutral detergent fiber 10.3%, acid detergent fiber 5.93%, ash 7.0%, and ether extract 2.2%.

### Sampling and measurements

Feed offered and refused was weighed and recorded daily for each individual animal for the duration of the experiment. DM content of the PMR offered and refused was determined daily to calculate daily DMI. Samples of GS, concentrate ration, barley straw, and GreenFeed bait were collected weekly and stored at −20 °C for subsequent chemical analysis. Feed samples were dried at 55 °C for 48 h in a forced-air oven and ground in a Lab Mill (Christy Turner, Suffolk, UK) through a 1 mm sieve for analysis. GS, PMR, concentrate ration, and GreenFeed bait were composited (equal DM weight) in 3-wk periods.

During this experiment, samples from the control and 3-NOP PMR that the animals were fed and prepared using the premix were collected daily, pooled by 3-wk periods frozen at −20 °C, and transported to DSM Nutritional Products (Kaiseraugst, Switzerland) on dry ice and analyzed for their 3-NOP content as described by Van Gastelen et al. (2020).

Body weight was recorded weekly (TRU-TEST, XR5000 weighing system; Tru-Test Group, Auckland, New Zealand) prior to feeding. On three separate occasions *viz.,* end of covariate period (week 0), week 6, and week 12, rumen fluid and digesta were collected from each animal, before feeding using a trans-esophageal rumen-sampling device. Feed was restricted from animals for a minimum of 2 h prior to sampling. After collection, rumen fluid pH was measured immediately using a digital pH meter (Orion SA 720; Thermo Fisher Scientific, Waltham, MA). Samples of rumen fluid (4 mL) were collected using an automatic pipette and mixed with 1 mL trichloroacetic acid (TCA; 500 g L^−1^, w/v) and stored at −20 °C for subsequent volatile fatty acids (VFA) and ammonia (NH_3_) analysis.

### Gaseous emissions

Enteric CH_4_, H_2_, and CO_2_ measurements were obtained on all animals using the GreenFeed emissions monitoring system (GEM; C-Lock Inc., Rapid City, SD) during the covariate period and the entire 12-wk experimental period. Throughout the study, four GEM systems were used, one assigned to each pen (17 animals per GEM unit), with gates positioned either side of the GEM system to ensure that only a single animal could access the system at any given time. Animals were adapted to the GEM system 2 wk prior to the start of the covariate period. A detailed description of the workings of the GEM has been previously described (Hammond et al., 2016). Animals were free to visit the GEM system throughout the day but were restricted to a maximum of three visits daily, with a 7 h interval between each visit, set to a maximum of 6 drops (40-s interval between drops) permitted per visit. This was to prevent the animals from consuming a large proportion of their daily DMI from the GreenFeed pellet. For each GEM system, the weight of 10 feed drops was recorded weekly with the average drop across all units 32.25 ± 2.25 g. The average number of daily drops of GreenFeed pellet was 15.3 drops d animal^−1^ throughout the CH_4_ measurement period and ranged from 10.1 to 21.2 drops d animal^−1^. The GreenFeed pellet utilized to entice animals to use the GEM was the same pelleted concentrate included in the PMR. All four GEM systems were equipped with both span and zero gas auto calibration systems, with auto calibrations performed every 3 d. The span gas contained CO_2_ 0.5%, CH_4_ 0.05%, H_2_ 0.001%, oxygen (O_2_) 21%, with the balance zero grade nitrogen gas (N_2_); (BOC Gas, Dublin, Ireland), and zero gas N_2_; (BOC Gas, Dublin, Ireland). Throughout the experiment, CO_2_ recovery tests were performed monthly as per the manufacturer’s instructions, to assess the airflow of the unit, with average CO_2_ recoveries of 99.22 ± 3.11%.

### Chemical analysis

The DM content of samples was determined after drying overnight at 105 °C (minimum 16 h) (method 930.15; AOAC International, 2005). Ash concentrations were determined by complete combustion in a muffle furnace (Nabertherm Gmbh, Lilienthal, DE) at 550 °C for 5 h (AOAC International, 2005). Starch was determined using the Megazyme Total Starch Assay Procedure (Product no: K-TSTA; Megazyme International Ireland LTD, Wicklow, IE; McCleary et al., 1994). The nitrogen content was determined using a LECO FP 528 instrument (Leco Corp, St. Joseph, MI, USA; AOAC International, 2005).

Neutral detergent fiber (NDF) and acid detergent fiber (ADF) were determined by the method of Van Soest et al. (1991) adopted for the use in the ANKOM 220 Fibre Analyzer (ANKOM Technology, NY, USA). Concentrate samples were analyzed with a thermos-stable α-amylase and 20 g of sodium sulfite was added to neutral detergent solution (NDS), while GS and incubation residues were analyzed with NDS only. NDF and ADF are expressed inclusive of residual ash. Gross energy of feed samples was determined by bomb calorimetry (Parr 1281 Bomb Calorimeter, Parr Instrument Company, IL, USA). Ether extract was determined using Soxtex instruments (Tecator, Hoganas, SE) and light petroleum ether in feed samples only.

Rumen liquor samples were thawed for 16 h at 4 °C and centrifuged at 1,800 × *g* for 10 min at 4 °C. One milliliter of supernatant was drawn off and diluted 1 in 5 with dH_2_O and centrifuged at 1,800 × *g* for 15 min at 4 °C. From this, 200 µL supernatant was drawn off and NH_3_ concentrations were determined using the phenolyhpochlorite method of Weatherburn (1967). For VFA analysis, a sample containing 250 μL of supernatant was drawn off into a separate test tube and diluted with 3.75 mL of dH_2_O and 1 mL of internal standard (0.5 g 3-methyl-*n*-valeric acid in 1 L of 0.15 M oxalic acid). Following centrifuging for 5 min, 260 × *g* at 21 °C, a subsample was filtered through a 0.45-μm filter (Cronus Syringe filter PTFE 13 mm; SMI-LabHut Ltd., Maisemore, Gloucester, UK) into a 4-mL gas chromatograph (GC) vial (Thermo Scientific, Langerwehe, Germany) and frozen at −20 °C until VFA analysis. One microliter of sample was injected via an auto sampler on a Varian GC 3800 with a 25 m × 0.53 mm i.d. megabore column (coating CP-Wax 58 (FFAP)—CB (no. CP7614), (Varian, Middelburg, the Netherlands)). The initial injector temperature was 75 °C, rising immediately to 95 °C, the temperature then increased at a rate of 3 °C min^−1^ until the temperature reached 200 °C, and was then held for 50 s. Nitrogen was used as a carrier gas. The pressure of the column was held at 2.3 psi and the column rate was 8.1 mL min^−1^.

### Statistical analyses

Data were analyzed using the PROC MIXED procedure of Statistical Analysis Software (SAS v9.4, Inst. Inc., Cary NC, USA). Normal distribution and homogeneity of variance were analyzed using the UNIVARIATE procedure. Data that were not normally distributed were transformed by raising the variable to the power of lambda. The appropriate lambda value was obtained by conducting a Box-Cox transformation analysis using the TRANSREG procedure of SAS. Performance data (start and final BW, total weight gained, average daily gain [ADG], and G:F) were analyzed using a model that included animal, block, and treatment. Emission variables and intakes from PMR and GEM, and GEM visits were reported at 3-wk intervals and analyzed as repeated measures. Data with repeated measures (gaseous emissions, rumen fermentation, intakes from PMR and GEM, and GEM visits) were analyzed for repeated measures. The model contained the same fixed effects as before, except covariate period and time-point were included as a fixed effect and treatment × time-point interaction. Since animals were pair-blocked within each pen, the block was included as a random effect in the model. For each variable, analyzed data were subjected to the following covariate structures: unstructured (UN), variance components (simple), compound symmetry (CS), heterogeneous CS, first-order autogressive (AR(1)), and heterogeneous (ARH(1)). The covariance structure that yielded the smallest Schwarz’s Bayesian criterion value was considered the most desirable for analysis. Statistically significant differences between least squares means were tested using the PDIFF command. Effects were considered significant at *P* ≤ 0.05 and a tendency toward significance was considered at a value of *P* > 0.05 to *P* < 0.10.

## Results

### 3-Nitrooxypropanol concentration analysis

To account for feed intake from the GreenFeed, the target concentration in PMR was set at 156 mg kg^−1^ DM of total DMI, assuming a 4% DMI from the GreenFeed. In total, four different composite samples of PMR were analyzed (Table 2) from the 3-NOP group and four composite samples from the control group, thus covering the PMR fed throughout the study. Results show that there was no 3-NOP in the control PMR and that on average, for the 3-NOP PMR, the actual measured concentration was 153.8 mg kg^−1^ DMI in the PMR. Final analysis of the intake data showed that on average intake from GreenFeed was higher than expected, making up 7.6% of the total DMI rather than 4% as previously estimated when the study was set up. When accounting for this, due to the fact the bait did not contain any 3-NOP, the final received dose rate was calculated as 142 mg kg^−1^ DMI.

**Table 2.** Target and measured concentration of 3-nitrooxypropanol in PMR offered to young growing beef cattle on a 50:50 forage:concentrate diet

Item	Time-point 1		Time-point 2		Time-point 3		Time-point 4	
	Control^1^	3-NOP^1^	Control^1^	3-NOP^1^	Control^1^	3-NOP^1^	Control^1^	3-NOP^1^
Target concentration in PMR^2^ diets, mg 3-NOP kg^−1^	0	156	0	156	0	156	0	156
Measured concentration in PMR^2^ diets, mg 3-NOP kg^−1^	0	138.3	0	175.8	0	156.5	0	144.4
Recovery, %	0	88.6	0	112.7	0	100.3	0	92.6

^1^PMR samples from Control and 3-NOP treatments were collected throughout the study, frozen at−20 °C, and transported to DSM Nutritional Products (Kaiseraugst, Switzerland) on dry ice and analyzed for their 3-NOP content as described by Van Gastelen et al. (2020).

^2^PMR, partial mixed ration.

### DMI and animal performance

The effects of 3-NOP on DMI and animal performance are presented in Table 3. There was no difference observed between 3-NOP and CON treatments for total DMI, total BW gained, final BW, ADG, and feed efficiency (G:F) over the 12 wk of this experiment (*P* > 0.05). Total DMI consisted of PMR DMI and GreenFeed bait DMI (feed received during GreenFeed visits), and when PMR and GreenFeed bait DMI were analyzed separately, there was no difference between treatments (*P* > 0.05). The daily number of visits to the GEM did not differ between treatment groups (*P* > 0.05) during the 12-wk study. However, there was a period effect, whereby mean daily visitations decreased (*P* < 0.001) at time-points 3 and 4 (weeks 7 to 12) compared to time-points 1 and 2 (weeks 1 to 6).

**Table 3.** The effect of 3-nitrooxypropanol (3-NOP^1^) supplementation on DMI, GreenFeed visits, gain:feed, and growth performance in young growing beef cattle offered a 50:50 forage:concentrate diet

Item^2^	Treatment		SEM	*P*-value		
	Control	3-NOP		Treatment	Time-point	Interaction
Total DMI, kg d^−1^	6.31	6.19	0.157	0.577	<0.001	0.357
PMR DMI, kg d^−1^	5.86	5.73	0.158	0.579	<0.001	0.321
GreenFeed bait, kg d^−1^	0.46	0.45	0.010	0.615	<0.001	0.480
GreenFeed visits	3.00	2.98	0.055	0.777	<0.001	0.492
Initial BW, kg	190.0	189.3	5.84	0.667	—	—
Final BW, kg	308.7	308.2	7.66	0.890	—	—
Total weight gained, kg	119.4	118.2	2.93	0.737	—	—
ADG, kg	1.42	1.41	0.035	0.737	—	—
Gain:feed	0.23	0.23	0.005	0.638	—	—

^1^3-NOP was offered at 142 mg kg^−1^ DM.

^2^DMI, dry matter intake; PMR, partial mixed ration; GreenFeed bait, dry matter intake from GreenFeed bait; BW, body weight; ADG, average daily gain.

### Rumen fermentation parameters

The effects of 3-NOP on rumen fermentation parameters are presented in Table 4. Animals that were offered 3-NOP had a higher ruminal pH (6.75) compared to the animals in the control treatment (6.62; *P* < 0.001). Ruminal NH_3_ concentrations were lower in animals offered 3-NOP (*P* < 0.001).

**Table 4.** The effect of 3-nitrooxypropanol (3-NOP^1^) supplementation on ruminal fermentation parameters in young growing beef cattle offered a 50:50 forage:concentrate diet

Item	Treatment		SEM	*P*-value		
	Control	3-NOP		Treatment	Time-point	Interaction
pH	6.62	6.75	0.035	<0.001	0.388	0.580
Concentration, mmol L^−1^						
NH_3_	3.73	3.05	0.154	<0.001	0.232	0.948
Acetate	63.45	61.80	2.491	0.628	0.620	0.797
Propionate	17.72	18.61	0.904	0.443	0.507	0.401
Butyrate	15.22	16.05	0.700	0.350	0.533	0.290
Valerate	1.37	1.39	0.073	0.909	0.212	0.575
Iso-butyrate	1.10	1.04	0.061	0.479	0.232	0.606
Iso-valerate	2.05	2.29	0.097	0.087	0.429	0.303
Total VFA	101.4	101.9	4.20	0.928	0.678	0.790
Ac:Pr	3.73	3.46	0.116	0.075	0.294	0.322

^1^3-NOP was offered at 142 mg kg^−1^ DM.

Concentrations of ruminal total VFA and individual VFA concentrations were not affected by the inclusion of 3-NOP (*P* > 0.05). Iso-valerate concentrations tended to be higher for animals that received 3-NOP (*P* < 0.10). Furthermore, acetate:propionate (Ac:Pr) tended to be lower with animals that received 3-NOP (*P* < 0.10).

### Gaseous emissions

**Figure 1.**
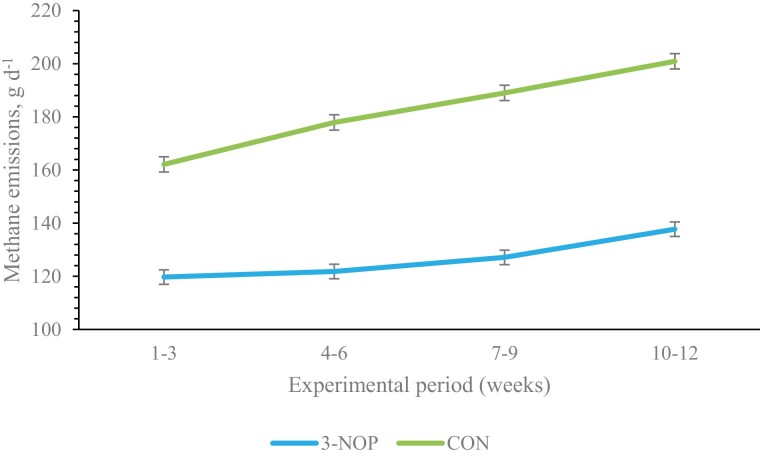
The effect of 3-NOP supplementation on methane emissions (CH_4_, g d^−1^) in growing beef cattle (≤6 mo) that were offered a 50:50 forage:concentrate diet over the course of the experiment. Treatments consisted of basal diet, control (CON), and basal diet plus 142 mg kg^−1^ DM of 3-NOP. Methane emissions were measured using GreenFeed measuring system (C-Lock Inc., Rapid City, SD). Data are LSM and error bars represent SEM.

**Figure 2.**
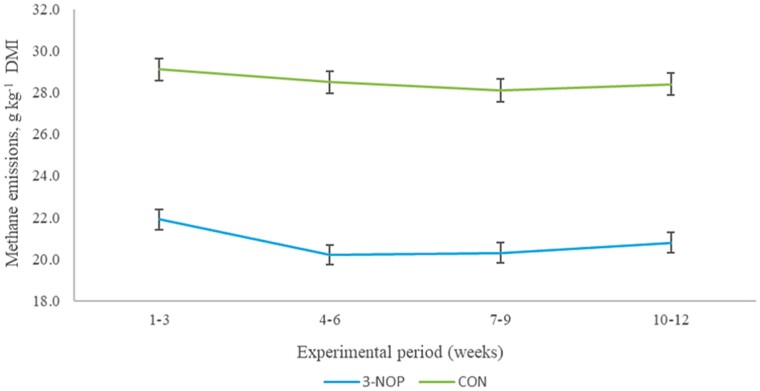
The effect of 3-NOP supplementation on methane emissions (CH_4_, g kg^−1^ total DMI) in growing beef cattle (≤6 mo) that were offered a 50:50 forage:concentrate diet over the course of the experiment. Treatments consisted of basal diet, control (CON), and basal diet plus 142 mg kg^−1^ DM of 3-NOP. Methane emissions were measured using GreenFeed measuring system (C-Lock Inc., Rapid City, SD). Data are LSM and error bars represent SEM.

The effects of 3-NOP on gaseous emissions are presented in Table 5. There was a treatment × time-point interaction for CH_4_ g d^−1^ (*P* < 0.001). Methane emissions (g d^−1^) increased over time in the Control cattle; the reductions (CH_4_ g d^−1^) observed with animals offered 3-NOP were different between the different time-points and were greater over time. Gaseous emissions: CH_4_ (g d^−1^, g kg^−1^ BW); H_2_ g d^−1^, and CO_2_ kg d^−1^ increased over time (*P* < 0.001). The inclusion of 3-NOP at 142 mg kg^−1^ DM in the basal diet decreased daily CH_4_ production (g d^−1^) by 30.6% (*P* < 0.001) and persisted over the course of the experiment (Fig. 1). Similarly CH_4_ yield (g kg^−1^ total DMI) was reduced by 27.2% (*P* < 0.001; Fig. 2), and CH_4_ intensity (g kg^−1^ BW d^−1^) was 30.2% lower when 3-NOP was included in the basal diet. Moreover, H_2_ production (g d^−1^) and yield (g kg^−1^ total DMI) increased by 227% and 205%, respectively, as a result of 3-NOP inclusion (*P* < 0.001), while there was no difference between dietary treatments for CO_2_ production (g d^−1^) and yield (g kg^−1^ total DMI) (*P* > 0.05).

**Table 5.** The effect of 3-nitrooxypropanol (3-NOP^1^) supplementation on gaseous emissions in young growing beef cattle offered a 50:50 forage:concentrate diet

Item^2^	Treatment		SEM	*P*-value		
	Control	3-NOP		Treatment	Time-point	Interaction
CH_4_, g d^−1^	182.5	126.6	2.36	<0.001	<0.001	<0.001
CH_4_, g kg^−1^ total DMI	28.6	20.8	0.381	<0.001	<0.01	0.380
CH_4_, g kg^−1^ BW d^−1^	0.76	0.53	0.010	<0.001	<0.001	0.060
H_2_, g d^−1^	1.12	3.67	0.096	<0.001	<0.001	0.567
H_2_, g kg^−1^ total DMI	0.20	0.61	0.025	<0.001	0.858	0.168
CO_2_, kg d^−1^	5.65	5.69	0.056	0.421	0.391	0.203
CO_2_, kg kg^−1^ total DMI	0.922	0.943	0.0195	0.423	0.391	0.203

^1^Average concentration of 3-NOP during the study was 142 mg kg^−1^ DM.

^2^DMI, dry matter intake was reported as total intake during the experiment, intake derived from partial mixed ration, and intake from GreenFeed pellet.

## Discussion

Enteric CH_4_ mitigation strategies based on dietary manipulations in ruminant feeding systems such as the inclusion of feed additives are being extensively studied globally in order to reduce the ruminal CH_4_ emissions from the animal production systems (Beauchemin et al., 2020; Bharanidharan et al., 2021; Hegarty et al., 2021) and assist in meeting international targets to reduce agri-GHG emissions. While 3-NOP has been proven effective in adult ruminants, the current study focused on evaluating the effects of its inclusion on young growing beef cattle when offered a 50:50 forage:concentrate diet.

### DMI and animal performance

The present study confirmed that the inclusion of 3-NOP in growing cattle diets is an effective strategy for reducing enteric CH_4_ emissions. In addition, the observed reductions in CH_4_ emissions were not associated with any negative effects on DMI and any animal performance parameters (total BW gain, ADG, G:F) measured. The effect of 3-NOP on DMI varies across studies which may be explained by feeding level offered, animal type, and the duration of the study (Kim et al., 2020). Feed efficiency was not affected by the inclusion of 3-NOP in the current study. Previous studies evaluating the efficacy of 3-NOP in beef cattle-fed forage diets demonstrated no negative effects on DMI (Romero-Perez et al., 2015; Martinez-Fernandez et al., 2018). In contrast, Vyas et al. (2016) offered two feeding levels of 3-NOP (100 mg kg^−1^ DM and 200 mg kg^−1^ DM) and only observed reductions in DMI when 3-NOP was offered at 200 mg kg^−1^ DM. Similarly, Zhang et al. (2021) noted reductions in DMI when 3-NOP was offered at 200 mg kg^−1^ DM. The results from the aforementioned studies are at odds with Martinez-Fernandez et al. (2018) where 3-NOP offered to steers at 330 mg kg^−1^ DM consuming an ad libitum forage diet increased DMI. An important factor to consider when using 3-NOP is the method by which the compound is incorporated into the diet (Kim et al., 2019). Indeed, Hristov et al. (2015) concluded that 3-NOP is effective in decreasing enteric CH_4_ emissions provided the additive is continuously present in the rumen, which is possible when it is mixed in a total mixed ration and, hence, intake is spread throughout the day. Therefore, this strategy can be applied when cattle are indoors, typically during the winter period of pastoral production systems.

### Rumen fermentation

The reduction in CH_4_ emissions by the inclusion of 3-NOP was associated with an increase in rumen pH, and based on a number of meta-analyses, it is expected that rumen pH would be higher in animals supplemented with 3-NOP (Dijkstra et al., 2018; Jayanegara et al., 2018; Kim et al 2020). In studies involving dairy cows, rumen pH increased when 3-NOP was pulse-dosed into the rumen (Reynolds et al., 2014) or offered through a total mixed ration (Melgar et al., 2020a). The increase in ruminal pH in the aforementioned studies can be explained by the observed decreases in total VFA concentrations. Similarly, reductions in DMI or changes in feeding frequency behavior, can lead to decreased concentrations of total VFA and a shift in the VFA fermentation profile. An increase in butyrate synthesis in the rumen is associated with an increase in ruminal pH due to the removal of an additional proton (Penner et al., 2009). In the current study, DMI was only numerically lower and feeding frequency was not measured. The only significant changes observed in the fermentation profiles were an increase in rumen pH and a decrease in NH_3_ concentration, with a tendency to increase iso-valerate concentrations and reduce Ac:Pr. In previous studies, 3-NOP inclusion resulted in the concentration/proportion of acetate decreasing while increasing propionate, butyrate, and valerate concentrations/proportions were detected. However, in the current study, the concentrations of these ruminal VFA were numerically different, portrayed by the tendency for lower Ac:Pr observed with animals that received the 3-NOP treatment compared to the control animals. Another possible explanation may be a reduction in lactate which has a low p*K*_a_ and high acidotic potential compared with other VFA produced within the rumen. However, this is only speculation as lactate was not measured in the current study, but the conversion of lactate into propionate via the acrylate pathway could explain the numerical increase in ruminal propionate concentrations. Nonetheless, these findings should be treated with caution and may be a reflection of the sampling protocol rather than a true reflection of the treatments. Rumen samples were collected using a trans-esophageal rumen-sampling device. Possible saliva contamination of the samples would lead to increased pH values. However, all animals were sampled using the same technique; therefore, differences in pH are expected to be a treatment effect and not due to potential saliva contamination. Moreover, samples were collected on three separate occasions, with animals off feed 2 h prior to sampling. As the compound is soluble and quickly metabolized in the rumen, it can be assumed that its impact on rumen fermentation at that point was diminished. This was unlike previous studies (Reynolds et al., 2014; Romero-Perez et al., 2014; Melgar et al., 2020a) where cannulated cattle were used and samples collected at different time-points post feeding via the cannula.

While the inclusion of 3-NOP in the diets fed to beef cattle in this study resulted in a reduction in ruminal NH_3_ concentrations, the levels observed were adequate for microbial synthesis and fiber digestion (Kang-Meznarich and Broderick, 1980). Similar observations were reported by Schilde et al. (2021) in dairy cows fed 3-NOP and supported in the meta-analysis by Jayanegara et al. (2018). Increases in ruminal butyrate concentrations stimulate blood flow and NH_3_ absorption from the rumen (Rémond et al., 1993) which may explain the reductions in ruminal NH_3_ observed by Lopes et al. (2016) and Schilde et al. (2021). However, in this study, while butyrate concentrations were numerically higher for the animals that received 3-NOP, they were not significant which may be a reflection of time at which the samples were collected relative to feeding (Allen, 1997), which was 2 h prior to feeding in the current study compared to 3 to 4 h post feeding in the previous studies.

### Gaseous emissions

Methane mitigation was achieved from the time 3-NOP was introduced into the animal’s diet and remained persistent throughout the 12-wk study. The increase in CH_4_ emissions over the course of the study was to be expected in young growing animals. During the supplementation period, the reduction in daily CH_4_ emissions (g d^−1^) and CH_4_ yield (g kg^−1^ DMI) was greater over time with an overall average reduction of 30.6% and 27.2%, respectively, in animals receiving 3-NOP, which is in agreement with previous long-term studies where beef (Vyas et al., 2016; Alemu et al., 2021) and dairy (Van Wesemael et al., 2019; Melgar et al., 2021) cattle diets were supplemented with 3-NOP. The CH_4_ yield observed in the current study was higher compared to animals receiving similar diets in previous studies (Zhang et al., 2021) influenced by a number of factors (Cottle and Eckard, 2018). While the efficacy of 3-NOP in reducing CH_4_ emissions in beef cattle has been confirmed in many previous studies (Romero-Perez et al., 2015; Vyas et al., 2016; Kim et al., 2019; Alemu et al., 2020, 2021; Melgar et al., 2020c), to our knowledge, this is the first study to evaluate the effectiveness of 3-NOP in reducing CH_4_ emissions in young growing cattle (<6 mo, supplemented for period of 12 wk) without affecting DMI and growth rate (Table 3). A meta-analysis across 11 experiments, involving dairy and beef cattle, summarized the effects of 3-NOP and found an average dose of 3-NOP (123 mg kg^−1^ DM) in beef cattle reduced CH_4_ production by 22.2% and CH_4_ yield by 17.1%. Additionally, for every 10 mg kg^−1^ DM increase in the inclusion level from the mean (123 mg kg^−1^ DM), a decrease in CH_4_ production and CH_4_ yield by 2.6% and 2.5%, respectively, would be expected (Dijkstra et al., 2018). The authors of the aforementioned meta-analysis also indicated that the effectiveness of 3-NOP in reducing CH_4_ emissions was negatively associated with the NDF content of the diet, and for every 10 g kg^−1^ increase in the NDF content of the diet from the mean of 331 g kg^−1^ of DM reduced the effectiveness of 3-NOP on CH_4_ production by 1.6% and the effect on CH_4_ yield by 1.5%. The reduction in CH_4_ production and yield observed in this study (30.6% and 27.2%, respectively) is consistent with these findings given that the inclusion level was 142 mg kg^−1^ DM and the mean NDF content of the diet was 335.4 g kg^−1^ DM.

During the dominant methanogenesis pathway (hydrogenotrophic methanogenesis), H_2_ is oxidized to H^+^, and CO_2_ is reduced to CH_4_ (Lyu et al., 2018; Smith et al., 2022). The impediment of rumen methanogenesis leads to a build-up of H_2_ in the rumen (Trei et al., 1972). An increase in H_2_ emissions with animals supplemented with 3-NOP is an indication of raised dissolved H_2_ in ruminal fluid (Melgar et al., 2020c), although this relationship is weak (Wang et al., 2016). If 4 mol of H_2_ is required to yield 1 mol of CH_4_, the 55.9 g d^−1^ or 3.5 mol d^−1^ average reduction in CH_4_ emissions (182.5 vs. 126.6 g d^−1^) as a result of the inclusion of 3-NOP in the current study would represent 14.0 mol d^−1^ H_2_ not being utilized for methanogenesis. Therefore, the increase in H_2_ emissions observed with animals offered 3-NOP in this study only represents a small proportion of the H_2_ not used for CH_4_ synthesis and may have been directed to alternative pathways. Alternative sinks for H_2_ include particular VFA (propionate, butyrate, and valerate), formate, ethanol, microbial mass (microbial long-chained fatty acids), and dissolved H_2_ pool in the rumen fluid (Ungerfeld, 2020). As the aforementioned variables were not measured in the current study, the higher H_2_ emissions observed in animals offered 3-NOP do not explain the fate of the energy spared by CH_4_ mitigation.

## Conclusion

Offering 3-NOP effectively reduced CH_4_ emissions in young growing beef cattle when included at 142 mg kg^−1^ DM and was persistent over the course of the 12-wk study, with greater reductions observed over time. The reductions in CH_4_ emissions observed are consistent with those reported in the literature for beef and dairy cattle. While there were differences in rumen fermentation patterns noted, DM feed intake and animal performance were not affected by the inclusion of 3-NOP, indicating that the inclusion of 3-NOP did not affect the organoleptic properties of the diet. This is the first study to demonstrate the reduction in CH_4_ emissions by 3-NOP in beef cattle at 6 mo of age offered a 50:50 forage:concentrate diet.

## Abbreviations

Ac:Pr: acetate:propionate
ADF: acid detergent fiber
ADG: average daily gain
BW: body weight
CH_4_: methane
CO_2_: carbon dioxide
dH_2_O: distilled water
DM: dry matter
DMI: dry matter intake
G:F: gain:feed
GEM: GreenFeed emission monitoring
GHG: greenhouse gas
GS: grass silage
H_2_: hydrogen
N_2_: nitrogen gas
NaSO_3_: sodium sulfite
NDF: neutral detergent fiber
NDS: neutral detergent solution
NH_3_: ammonia
O_2_: oxygen
PMR: partial mixed ration
SiO_2_: silicone dioxide
VFA: volatile fatty acids
3-NOP: 3-nitrooxypropanol
